# Current and emerging strategies for the management of brain and leptomeningeal metastases in melanoma

**DOI:** 10.3389/fonc.2026.1874139

**Published:** 2026-09-22

**Authors:** Safa Idoudi, Karine Billard, Emilien Ezine, Marie Fournier, Nicolo Norri, Philippe Decq, Stephane Goutagny, François Nataf, Celeste Lebbé, Matthieu Faillot

**Affiliations:** 1 AP-HP Dermato-oncology and Centre d'Investigation Clinique (CIC), Cancer Institute APHP, Saint Louis Hospital, Paris, France; 2 Department of Neurosurgery, AP-HP, Beaujon Hospital, Clichy, France; 3 Université Paris Cité, Paris, France; 4 Georges Charpak Institute of Human Biomechanics, École Nationale Supérieure des Arts et Métiers (ENSAM), Paris, France; 5 Department of Radiation Oncology, Saint-Louis Hospital (AP-HP), Paris, France; 6 Institut National De la Santé Et De la Recherche Médicale (INSERM), Paris, France; 7 Biomaps Research Laboratory, Commissariat Á l’Énergie Atomique Et Aux Énergies Alternatives (CEA), Orsay, France

**Keywords:** brain metastases, immunotherapy, intrathecal delivery, melanoma, radiotherapy, targeted therapy

## Abstract

Melanoma exhibits a marked predilection for central nervous system (CNS) dissemination, including both brain parenchymal metastases and leptomeningeal metastases (LMM), which together represent a major cause of morbidity and mortality. Historically characterized by poor prognosis, CNS melanoma has undergone a paradigm shift in management over the last decade with the advent of immune checkpoint inhibitors and targeted therapies, as well as advances in local treatment modalities such as stereotactic radiosurgery. While outcomes for patients with melanoma brain metastases have significantly improved, leptomeningeal melanoma metastases remain a devastating condition with limited evidence-based therapeutic options. This review provides a comprehensive and up-to-date overview of the epidemiology, biological determinants, diagnostic strategies, and therapeutic management of melanoma with cerebral and leptomeningeal involvement. Particular emphasis is placed on systemic treatment strategies, including immune checkpoint inhibitors, BRAF/MEK-targeted therapies, and their integration with local treatments. Dedicated attention is given to leptomeningeal disease, highlighting emerging data on systemic immunotherapy, intrathecal approaches, and innovative therapeutic strategies aimed at overcoming the blood–brain and blood–CSF barriers. Finally, we address current challenges, unmet clinical needs, and future perspectives, including biomarker development, novel agents, and rational combination strategies, with the goal of improving outcomes for this high-risk population.

## Introduction

Cutaneous melanoma is one of the solid tumors with the highest propensity to metastasize to the central nervous system (CNS). Brain metastases occur in up to 40–50% of patients during the course of advanced disease, and autopsy series report CNS involvement in up to 70% of cases (1). In addition to parenchymal brain metastases, melanoma is among the malignancies most frequently associated with leptomeningeal metastases (LMM), a particularly aggressive form of CNS dissemination characterized by diffuse infiltration of the leptomeninges and cerebrospinal fluid (CSF) (2).

Until recently, the diagnosis of CNS involvement in melanoma was associated with extremely poor survival, often measured in weeks to a few months, especially in patients with leptomeningeal disease. Therapeutic options were limited to palliative whole-brain radiotherapy and corticosteroids, with minimal impact on long-term outcome (3).

However, the therapeutic landscape has profoundly evolved over the last decade. Immune checkpoint inhibitors (ICIs), particularly the combination of anti–CTLA-4 and anti–PD-1 antibodies, and BRAF/MEK inhibitors for BRAF-mutant melanoma have demonstrated intracranial activity, reshaping the prognosis of selected patients with melanoma brain metastases (4).

Despite these advances, several critical challenges remain. Patients with symptomatic brain metastases, those requiring corticosteroids, and individuals with leptomeningeal disease are underrepresented in pivotal clinical trials. Moreover, CNS-specific response patterns under immunotherapy, including pseudoprogression and discordant intracranial versus extracranial responses, complicate disease assessment and therapeutic decision-making (5).

The aim of this review is to synthesize current knowledge on clinical management of melanoma CNS metastases, encompassing both brain parenchymal and leptomeningeal involvement. We focus on biological mechanisms, diagnostic approaches, and contemporary treatment strategies, while highlighting emerging therapies and future directions to address the persistent unmet clinical needs in this field.

## Epidemiology and clinical burden of CNS and leptomeningeal metastases in melanoma

### Incidence and risk factors

Melanoma is disproportionately represented among cancers that metastasize to the CNS (5). The cumulative incidence of brain metastases increases with disease duration and stage, particularly in patients with visceral involvement. Risk factors for CNS dissemination include advanced stage at diagnosis, high tumor burden, ulcerated primary tumors, and specific molecular features such as BRAF mutations, although data remain heterogeneous (2).

Leptomeningeal metastases occur less frequently than parenchymal brain metastases but represent a growing clinical challenge as systemic disease control improves and patient survival increases. Reported incidence rates of LMM in melanoma range from 5% to 15% in advanced disease, with melanoma accounting for a significant proportion of solid tumor–related leptomeningeal metastases (6, 7).

Brain metastases typically occur 2–4 years after diagnosis of the primary tumor. The median age at onset is 59 years, with a clear male predominance. Lesions are solitary in 40% of patients and multiple in 60%. Most metastases are supratentorial (72%), while 6% are infratentorial and 16% involve both compartments. Leptomeningeal involvement is observed in 11% of cases. Concomitant extra-cranial metastases are present in 80% of patients (2, 8–10).

### Biological determinants and molecular drivers of CNS tropism

The marked propensity of melanoma to colonize the central nervous system reflects both tumor-intrinsic genetic alterations and a permissive brain microenvironment. Oncogenic BRAF V600 and NRAS mutations, together with loss of the tumor suppressor PTEN, are recurrently enriched in brain-metastatic clones and converge on constitutive activation of the PI3K/AKT/mTOR axis, which supports tumor-cell survival and outgrowth within the brain parenchyma. Complete loss of PTEN expression has been correlated with a shorter interval to brain metastasis and reduced overall survival in stage III BRAF-mutant melanoma, supporting a biological link between this alteration and CNS dissemination (11).

Cross-talk with brain-resident cells is equally important. Reactive astrocytes secrete exosomes carrying PTEN-targeting microRNAs that reversibly silence PTEN in disseminated melanoma cells, while astrocyte-derived chemokines such as CCL17 engage the CCR4 receptor to favor homing to the brain. Activation of STAT3, neurotrophin signaling through CD271/p75NTR, and secretion of matrix metalloproteinases such as MMP-2 further facilitate transmigration across the blood–brain barrier and perivascular colonization. These mechanisms help to explain why the CNS can act as a sanctuary site, with intracranial progression sometimes occurring despite extracranial disease control, and they nominate the PI3K/AKT and neurotrophin pathways as candidate targets for CNS-directed therapeutic strategies (12).

### Prognostic implications

Historically, median overall survival for patients with melanoma brain metastases was approximately 4–6 months, and less than 2 months for those with leptomeningeal disease (3). The introduction of immune checkpoint inhibitors has markedly improved survival in selected patients with asymptomatic brain metastases, with median overall survival now exceeding two years in some series. In the modern era, patients with melanoma brain metastases diagnosed between 2015 and 2019 demonstrated a median overall survival of 13 months compared to 7 months for those diagnosed between 2010 and 2014, reflecting the impact of immune checkpoint inhibitors and targeted therapies (13).

In contrast, prognosis for LMM remains poor, with median survival typically reported between 8 and 16 weeks, although recent retrospective data suggest modest improvements in subsets of patients treated with modern immunotherapy.

### Clinical presentation

Non-specific manifestations include seizures in 11% of patients and other neurological symptoms, in more than 50%. Hydrocephalus was noted in 7% of cases. Haemorrhagic brain metastases are reported in approximately 35% of cases. Asymptomatic lesions are detected in about 30% of patients, usually identified through systematic magnetic resonance imaging (MRI) screening (2, 8–10).

Leptomeningeal metastases often manifest with multifocal and fluctuating neurological symptoms, including cranial nerve palsies, radiculopathies, altered mental status, and signs of increased intracranial pressure. The heterogeneity and nonspecific nature of symptoms frequently delay diagnosis (14).

On imaging, parenchymal melanoma metastases are frequently multiple and predominantly supratentorial, and often display intrinsic T1 hyperintensity related to melanin content or to subacute haemorrhage, with variable contrast enhancement and surrounding vasogenic oedema; susceptibility-weighted sequences typically disclose hemorrhagic foci (15). The principal differential diagnoses of an enhancing parenchymal lesion include haemorrhagic metastases from other primary tumours and cavernous malformation; after local treatment, radiation necrosis and pseudoprogression must also be considered. In leptomeningeal involvement, imaging may show smooth or nodular leptomeningeal and cranial-nerve enhancement, for which infectious or granulomatous meningitis and benign post-lumbar-puncture dural enhancement are the main alternative explanations (16).

## Diagnostic work-up and response assessment

### Neuroimaging

Contrast-enhanced brain magnetic resonance imaging (MRI) is the gold standard for the detection of melanoma brain metastases. Advanced MRI techniques, including susceptibility-weighted imaging and perfusion sequences, are particularly useful given the hemorrhagic propensity of melanoma metastases. The MRI protocol should include: 1) non-contrast T1, 2) diffusion, 3) T2* or susceptibility-weighted imaging, 4) dynamic susceptibility contrast perfusion when radiation necrosis is suspected, 5) T1 with contrast injection preferentially using the 3D spin echo images (17).

For leptomeningeal disease, MRI of the brain and entire spine with gadolinium is essential. Typical findings include leptomeningeal enhancement along cortical sulci, cranial nerves, spinal nerve roots, and the cauda equina. However, these abnormalities may appear linear or very focal, and imaging sensitivity remains imperfect, especially in early disease. Current guidelines support the use of high resolution MRI with 1 mm slices for brain imaging and 3 mm slices for spine imaging (18). Use of FLAIR sequences with contrast injection is suggested (18) as it seems more sensitive for the detection of leptomeningeal disease.

### Cerebrospinal fluid analysis

CSF cytology remains the diagnostic gold standard for leptomeningeal metastases but suffers from limited sensitivity, particularly with single lumbar punctures. Repeated sampling improves diagnostic yield. Emerging techniques, including CSF circulating tumor DNA, circulating tumor cells, and proteomic profiling, show promise for earlier diagnosis and disease monitoring (18).

Beyond cytology, routine CSF examination in suspected leptomeningeal disease commonly shows non-specific abnormalities, including raised opening pressure, elevated total protein, reduced glucose, and a lymphocyte-predominant pleocytosis; melanin-laden or overtly atypical cells are identified only in a minority of samples. Because a single cytological assessment detects fewer than half of cases, at least two to three large-volume lumbar punctures, with prompt processing, are recommended before leptomeningeal disease can be considered excluded. Flow cytometry and immunocytochemistry for melanocytic markers such as SOX10, MelanA/MART-1 and HMB-45 can increase diagnostic confidence when cytomorphology is equivocal. CSF-derived cell-free tumour DNA reflects the molecular profile and burden of leptomeningeal disease more reliably than plasma and, together with circulating tumour cell enumeration, is emerging as a sensitive compartment for early diagnosis and longitudinal monitoring (19).

### Response assessment and criteria

Conventional RECIST (Response Evaluation Criteria in Solid Tumours) criteria may be suboptimal for assessing CNS disease, particularly in the context of immunotherapy. The Response Assessment in Neuro-Oncology (RANO) criteria and their immunotherapy adaptation (iRANO) have been developed to better capture CNS-specific response patterns and pseudoprogression (20). For leptomeningeal disease, response assessment remains challenging, relying on a combination of clinical evaluation, MRI findings, and CSF analysis. Standardized response criteria for LMM are still evolving (21).

Under the RANO-BM framework, a measurable brain metastasis is defined as a contrast-enhancing lesion with a longest diameter of at least 10 mm and a perpendicular diameter of at least 5 mm, visible on two or more contiguous axial slices; up to five target lesions are followed and the CNS is scored as a compartment separate from extracranial disease (22). Best overall CNS response (complete response, partial response, stable disease, or progressive disease) is a composite of target-lesion measurements, non-target and new-lesion assessment, corticosteroid requirement, and clinical status, with confirmation of a complete or partial response at least four weeks later when CNS response is the primary endpoint. For leptomeningeal disease, the RANO-LM proposal integrates a standardized neurological examination, CSF cytology, and a structured radiographic scorecard into a composite response category, although its feasibility and clinical utility in routine practice remain debated. Adoption of these CNS-specific criteria, rather than RECIST alone, is important because intracranial and extracranial responses are frequently discordant and because immunotherapy-related pseudoprogression can be misclassified as true progression (23).

Current guidelines suggest performing an MRI every 2 to 3 months in patients with brain metastasis. Enlarging lesions are not always synonymous with disease progression, as they may reflect pseudoprogression related to immune checkpoint inhibitors or radiation necrosis in previously irradiated patients. Multimodal MRI, including perfusion imaging and spectroscopy, as well as molecular imaging techniques, may help differentiate tumor progression from radiation necrosis, although their diagnostic performance remains imperfect (24).

## Local therapies for melanoma brain and leptomeningeal metastases

### Surgery

Surgical resection remains an important component of the multidisciplinary management of melanoma brain metastases in carefully selected patients. Indications include solitary or limited brain metastases causing mass effect, symptomatic lesions refractory to corticosteroids, diagnostic uncertainty, or the need for rapid neurological decompression. Surgery is particularly beneficial in patients with good performance status, controlled extracranial disease, and accessible lesions (25).

However, the role of surgery has diminished with the increasing efficacy of stereotactic radiosurgery (SRS) and systemic therapies. In the era of immunotherapy and targeted therapies, surgery may also serve as a bridge to systemic treatment by rapidly alleviating symptoms and allowing corticosteroid tapering (26).In an emergency setting, particularly in cases of hemorrhage within a brain metastasis, rapid neurological deterioration requires urgent assessment. The decision to proceed with surgical intervention should ideally be made in close coordination with the oncology team responsible for the patient’s care. Key factors to consider include the patient’s preoperative performance status, the overall extent of intracranial and extracranial disease, and the availability and feasibility of systemic treatment options following surgery.

### Stereotactic radiosurgery

Stereotactic radiosurgery has become the cornerstone of local treatment for melanoma brain metastases. SRS delivers high-dose, focal radiation with excellent local control rates while sparing surrounding healthy brain tissue. Melanoma, historically considered radioresistant, responds well to high-dose per fraction radiation, making SRS particularly effective. Fractionated stereotactic radiosurgery (SRS) may be considered for larger lesions, typically those exceeding 3 cm in diameter (27).

A recent randomized study in patients resected for 1–3 brain metastases demonstrates that delivering SRS to the surgical cavity markedly decreases the risk of local relapse compared with simple postoperative observation (28).

Multiple prospective and retrospective studies have shown 1-year local control rates of around 80–90% for melanoma brain metastases treated with SRS, with rates highest for lesions below 2 cm in diameter and declining for larger volumes (29). It is also associated with increased OS (30) Importantly, SRS can be safely combined with systemic therapies, including immune checkpoint inhibitors and targeted therapies.

The combination of SRS and immune checkpoint inhibitors has emerged as a promising strategy for melanoma brain metastases. Retrospective studies suggest improved local control and overall survival compared to either modality alone, without a significant increase in neurotoxicity when appropriately timed (29). This potential synergistic effect between SRS and immunotherapy is possibly mediated through enhanced antigen presentation and immune activation (31).

The optimal sequencing of SRS and immunotherapy remains controversial. Some data suggest that concurrent or near-concurrent administration may enhance antitumor immunity through radiation-induced immunogenic cell death. However, prospective randomized trials are lacking (32).

Current practice favors SRS over whole-brain radiotherapy (WBRT) even in patients with multiple brain metastases, provided that the total tumor volume is limited and lesions are amenable to focal treatment. Fractionated stereotactic radiotherapy may be considered for larger lesions or those in eloquent brain regions to reduce the risk of radionecrosis (33).

It is not uncommon to detect small, asymptomatic brain metastases during routine follow-up imaging or alongside a larger, symptomatic lesion. Although multiple lesions can often be treated during a single stereotactic radiosurgery session, some of these smaller metastases may be effectively controlled with systemic immunotherapy and/or targeted therapies, making active surveillance a potential management option. This strategy may be particularly appropriate for lesions located in non-eloquent brain regions, where short-term growth is unlikely to cause neurological deterioration. However, there is currently no evidence or established guideline supporting active surveillance in place of systematic irradiation for newly detected metastases.

### Whole-brain radiotherapy

The role of WBRT in melanoma has significantly declined due to limited efficacy and substantial neurocognitive toxicity. Randomized trials in patients with melanoma brain metastases have failed to demonstrate a survival benefit with WBRT, either alone or as adjuvant therapy following surgery or SRS (34).

WBRT may still be considered in selected patients with extensive intracranial disease not amenable to SRS, poor performance status, or leptomeningeal involvement with diffuse nodular disease. However, in the era of effective systemic therapies, WBRT is rarely used, usually as a palliative option of last resort. When it is used, WBRT is most commonly delivered as 20 Gy in 5 fractions or 30 Gy in 10 fractions, and hippocampal-avoidance planning combined with memantine is now recommended to limit neurocognitive decline. In the dedicated phase III WBRTMel trial (ANZMTG 01.07), which randomized patients after local treatment of one to three melanoma brain metastases, the addition of adjuvant WBRT reduced the rate of intracranial relapse but did not improve overall survival, distant intracranial control at the level expected, or preservation of performance status, supporting the restriction of WBRT to selected palliative situations rather than routine adjuvant use (35).

#### Radiation necrosis

In a recent meta-analysis the estimated incidence of radionecrosis was 5%; however, this is variable, ranging from 6% of patients (36) to 18% of patients (17). The delayed nature of radionecrosis is being recognized with up to 22% of radionecrosis in patients surviving over a year after irradiation for brain metastasis (including patients with melanoma). Classical risk factors for radiation necrosis are lesion volume and radiation dose. In one monocentric retrospective study, BRAF V600 mutation was “protective” against radiation necrosis (37). Meta-analyses do not support immunotherapy as a risk factor for radiation necrosis. Management of symptomatic radiation necrosis includes corticosteroid therapy. Bevacizumab may be considered in patients without thromboembolic disease, high risk of intracranial hemorrhage, or recent surgery because of the risk of wound dehiscence. Surgical resection may be indicated in selected patients with symptoms refractory to medical therapy or when there is diagnostic uncertainty between radiation necrosis and tumor progression (38).

#### Hemorrhage in patients with MBM

MBM are at very high risk of bleeding (39). Whether non-cancer therapies such as oral anticoagulants or cancer-related therapies such as BRAF or MEK inhibitors or radiosurgery enhance bleeding risk remain uncertain (40).Oral anticoagulants seem to be associated with increased risk of ICH especially after radiosurgery (41). Pre-existing hemorrhage inside lesion and radiation dose seems also to be associated with risks of hemorrhage and is currently under investigation (42). Quantitatively, melanoma carries the highest risk of intratumoral haemorrhage among brain metastases of all histologies, with reported rates of roughly 30% to 50% of lesions, compared with under 5% for most epithelial primaries. Haemorrhage is graded according to the Common Terminology Criteria for Adverse Events and ranges from clinically silent microbleeds detected on susceptibility-weighted imaging to large symptomatic haematomas causing acute neurological deterioration; intratumoral haemorrhage is consistently associated with shorter survival. Reassuringly, symptomatic intracranial haemorrhage attributable to systemic therapy remains uncommon, occurring in approximately 2% of patients in the CheckMate 204 trial of nivolumab plus ipilimumab (4).

## Systemic therapies for melanoma brain metastases

### Immune checkpoint inhibitors

The advent of immune checkpoint inhibitors has revolutionized the treatment of melanoma brain metastases. Unlike conventional chemotherapy, ICIs have demonstrated clinically meaningful intracranial activity, even in the absence of prior local therapy. The principal trials of systemic therapy in melanoma brain and leptomeningeal metastases discussed below are summarized in **Table 1**.

**Table 1:** Selected pivotal trials of systemic therapy in melanoma brain and leptomeningeal metastases.

Trial (phase)	Regimen	Population	Key intracranial outcome
CheckMate 204 (II)	Nivolumab + ipilimumab	Active MBM; asymptomatic and corticosteroid-free (cohort A)	Intracranial ORR approximately 55%; sustained intracranial control in responders at long-term follow-up
ABC/Anti-PD1 Brain Collaboration (II)	Nivolumab ± ipilimumab vs Nivolumab alone	Asymptomatic MBM, with a separate cohort of symptomatic or previously treated MBM	Favors Nivo + Ipilimumab. At 7 years: intracranial ORR 51% vs 20%; intracranial PFS 42% vs 15%; OS 48% vs 26%.
COMBI-MB (II)	Dabrafenib + trametinib	BRAF V600-mutant MBM; four cohorts (locally treated or untreated, asymptomatic or symptomatic)	Intracranial ORR 44–59% across cohorts; median intracranial PFS approximately 5–7 months
E-BRAIN/GEM1802 (II)	Encorafenib + binimetinib, followed by optional sequential radiotherapy in responders	BRAF V600-mutant MBM; single-arm, n = 48 (25 asymptomatic, 23 symptomatic; corticosteroids permitted)	Intracranial ORR 70.8% at 2 months (80% in asymptomatic and 60.9% in symptomatic patients, *post hoc* subgroups); median intracranial PFS 8.5 months; median OS 15.9 months
SWOG S2000 (II, randomised)	Encorafenib + binimetinib + nivolumab vs ipilimumab + nivolumab	Symptomatic BRAF V600-mutant MBM (n = 30)	Favors Encorafenib + binimetinib + nivolumab. 6-month PFS 50% vs 29%; median PFS 6.2 vs 1.4 months (HR 0.51)
Brastianos et al. (II)	Nivolumab + ipilimumab (systemic)	Leptomeningeal metastases; single-arm	Feasible with acceptable safety; survival benefit observed in a subset of patients

MBM, melanoma brain metastases; ORR, objective response rate; PFS, progression-free survival; OS, overall survival; HR, hazard ratio. Outcomes are summarised from the respective publications cited in the text; values are not directly comparable across trials owing to differences in study design, populations and response criteria.

#### Anti–CTLA-4 and anti–PD-1 monotherapy

Early studies evaluating ipilimumab and anti–PD-1 agents (nivolumab, pembrolizumab) showed modest intracranial response rates, particularly in asymptomatic patients not requiring corticosteroids. Anti–PD-1 monotherapy generally demonstrated superior efficacy and tolerability compared to ipilimumab alone (43), with intracranial response rates in 26% of patients (44).

However, responses were often less durable than those observed extracranially (43), and outcomes remained poor in symptomatic patients or those receiving corticosteroids.

#### Combination of immunotherapy and targeted therapy

Combining immune checkpoint inhibitors with BRAF/MEK inhibitors represents another attractive strategy, aiming to leverage the rapid tumor control of targeted therapy with the durability of immunotherapy. Early-phase trials and retrospective studies have demonstrated encouraging efficacy but also increased toxicity (45).

A recent randomized phase 2 trial (SWOG S2000) in patients with symptomatic BRAF-mutant melanoma brain metastases demonstrated that frontline encorafenib plus binimetinib plus nivolumab achieved a 6-month progression-free survival rate of 50% compared to 29% with ipilimumab plus nivolumab, with a hazard ratio of 0.51 and median progression-free survival of 6.2 months versus 1.4 months. Prospective data specifically addressing combination strategies in CNS melanoma are still emerging (46, 47).

#### Combination immunotherapy: ipilimumab plus nivolumab

Combination therapy with ipilimumab and nivolumab represents the most effective systemic treatment for melanoma brain metastases to date. The pivotal CheckMate 204 trial demonstrated an intracranial objective response rate of approximately 55% in asymptomatic patients, with durable responses and prolonged overall survival (48). Three-year follow-up data confirmed sustained intracranial disease control in a substantial proportion of responders (4).

Similarly, the ABC trial confirmed superior intracranial response rates and progression-free survival with combination immunotherapy compared to nivolumab monotherapy (49). At 7-year follow-up, patients with asymptomatic brain metastases achieved an intracranial response rate of 51% with ipilimumab plus nivolumab versus 20% with nivolumab alone. The 7-year intracranial progression-free survival rate was 42% with combination therapy versus 15% with monotherapy, and overall survival at 7 years was 48% versus 26%, respectively (50).

Importantly, both trials highlighted a stark contrast in outcomes between asymptomatic and symptomatic patients, the latter exhibiting significantly lower response rates and survival. Combination immunotherapy is now considered the standard of care for patients with asymptomatic melanoma brain metastases who do not require corticosteroids (51, 52).

Toxicity remains significant, with high rates of immune-related adverse events, necessitating careful patient selection and multidisciplinary management (53).

## Targeted therapies for BRAF-mutant melanoma

Approximately 40–50% of melanomas harbor activating BRAF mutations, most commonly V600E or V600K. BRAF and MEK inhibitors have demonstrated rapid and clinically meaningful intracranial activity in this molecularly defined population (54).

The COMBI-MB trial evaluated dabrafenib plus trametinib in patients with BRAF-mutant melanoma brain metastases, including symptomatic patients and those with prior local therapy. Intracranial response rates ranged from 44% to 59%, with rapid tumor shrinkage and symptom improvement. However, responses were generally less durable than those observed with immunotherapy, with median intracranial progression-free survival of approximately 5–7 months (55).

In the E-BRAIN/GEM1802 phase 2 trial, encorafenib plus binimetinib was administered to patients with BRAF V600-mutant melanoma brain metastases, with sequential radiotherapy offered to those achieving a response or stable disease at the first tumour assessment. The intracranial objective response rate at 2 months was 70.8%, reaching 80% in asymptomatic and 60.9% in symptomatic patients in a *post hoc* subgroup analysis; median intracranial progression-free survival was 8.5 months and median overall survival 15.9 months. Treatment was generally well tolerated, with predominantly low-grade immune-related and BRAF/MEK-inhibitor–related toxicities and no treatment-related deaths (56).

Targeted therapy is particularly valuable in patients with symptomatic disease requiring rapid disease control or corticosteroids. However, long-term disease control remains challenging, and resistance inevitably develops (51).

### Sequencing and treatment selection

Optimal sequencing between immunotherapy and targeted therapy remains an area of active investigation. Retrospective analyses suggest that upfront immunotherapy may confer superior long-term survival compared to initial targeted therapy, even in BRAF-mutant patients, provided that disease burden and symptoms allow to wait for the efficacy of immunotherapy (57).

Current practice often favors upfront combination immunotherapy for asymptomatic patients without corticosteroid dependence, and targeted therapy for patients with rapidly progressive, symptomatic brain metastases or high steroid requirements, with consideration of subsequent immunotherapy upon disease stabilization (51). Prospective trials addressing sequencing strategies specifically in patients with brain metastases are ongoing. This preference for upfront immunotherapy is supported by extracranial randomized data: in the phase III DREAMseq trial, starting with nivolumab plus ipilimumab and switching to dabrafenib plus trametinib at progression produced superior 2-year overall survival (72% versus 52%) (57), compared with the reverse sequence, and the SECOMBIT trial showed favourable long-term survival with an immunotherapy-first or sandwich approach (58). In practical terms, candidate selection can be framed around a small number of variables: asymptomatic patients with limited disease burden, no corticosteroid requirement, and adequate performance status are generally offered upfront combination immunotherapy, whereas patients with bulky or rapidly progressive symptomatic disease, high steroid needs, or impending neurological compromise are started on BRAF/MEK inhibitors (when BRAF-mutant) to obtain rapid control, with immunotherapy introduced once disease and steroids have stabilized. Local therapy with SRS is integrated in parallel for measurable or symptomatic lesions. Because these brain-metastasis-specific sequencing questions have not been resolved prospectively, enrolment in dedicated trials remains the preferred option whenever feasible.

### Special considerations: corticosteroids and symptomatic disease

Corticosteroid use is a major negative prognostic factor in melanoma brain metastases. Steroids impair immune activation and are associated with reduced efficacy of ICIs. Whenever possible, strategies to minimize or rapidly taper corticosteroids are essential, including early use of local therapies or targeted agents (57).

Patients with symptomatic brain metastases remain a high-risk population with limited therapeutic options and poor outcomes, underscoring the need for innovative strategies and inclusion in clinical trials. The corresponding diagnostic and treatment algorithm for melanoma brain metastases is summarized in **Figure 1**.

**Figure 1:**
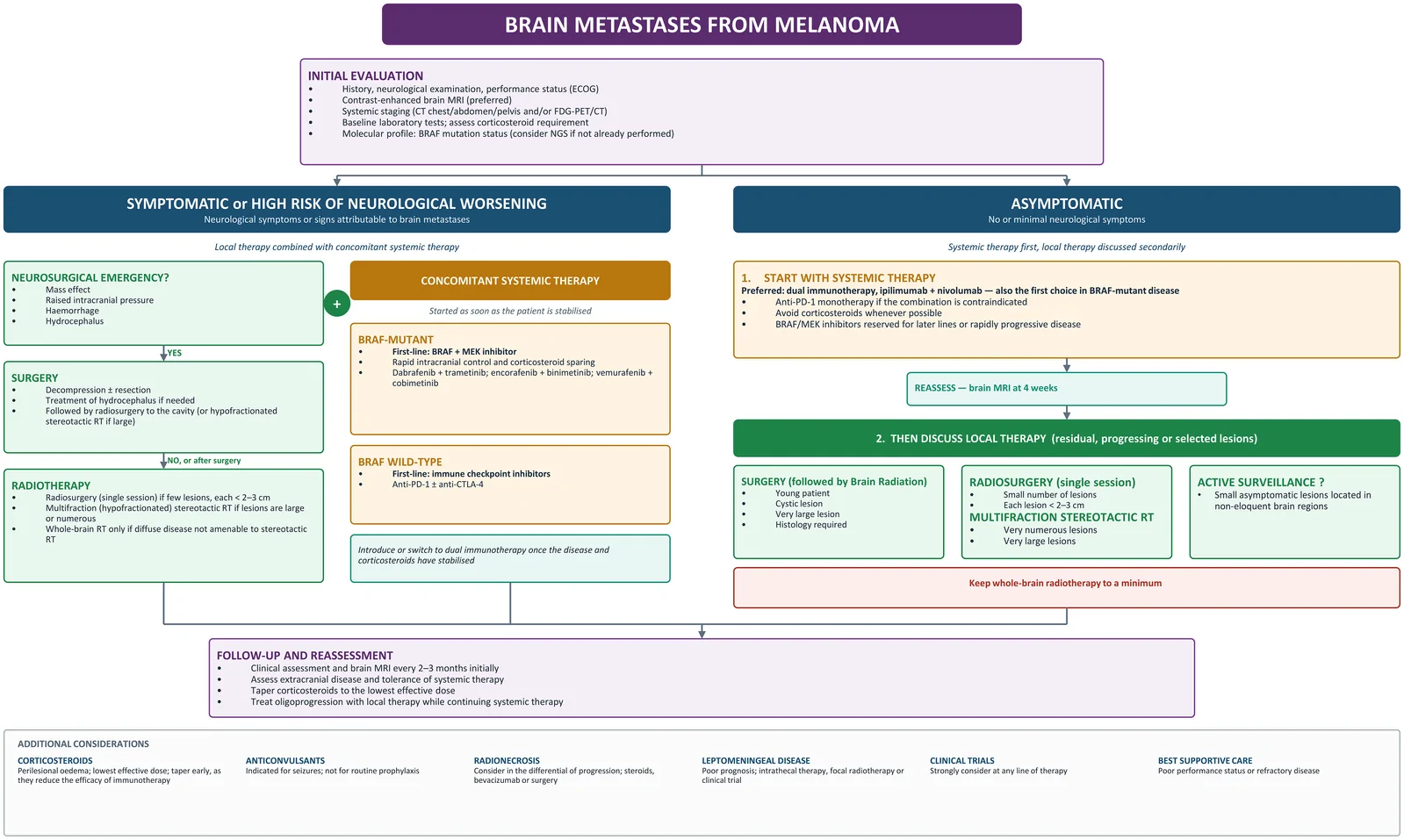
Diagnostic and treatment algorithm for melanoma brain metastases (BM), stratified by symptom status and BRAF mutation status. BM, brain metastases; BRAF, v-Raf murine sarcoma viral oncogene homolog B; ECOG, Eastern Cooperative Oncology Group; ICI, immune checkpoint inhibitor; NGS, next-generation sequencing; SRS, stereotactic radiosurgery; WBRT, whole-brain radiotherapy.

## Specific management of leptomeningeal melanoma metastases

### Clinical and therapeutic challenges

Leptomeningeal melanoma metastases (LMM) represent one of the most aggressive and therapeutically challenging manifestations of advanced melanoma. Unlike parenchymal brain metastases, LMM are characterized by diffuse dissemination of tumor cells within the cerebrospinal fluid (CSF) pathways, leading to multifocal neurological impairment and rapid clinical deterioration (54).

Patients with LMM are frequently excluded from clinical trials, resulting in a paucity of high-level evidence to guide management. Historically, median overall survival ranged from 4 to 8 weeks with best supportive care. Although modest improvements have been reported in the era of modern systemic therapies, LMM remains associated with dismal outcomes and a high symptom burden.

Major challenges include poor drug penetration across the blood–CSF barrier, rapid neurological decline, diagnostic delays, and the frequent need for corticosteroids, which may compromise immunotherapy efficacy.

## Systemic immunotherapy in leptomeningeal melanoma

### Immune checkpoint inhibitors

Emerging retrospective series and small prospective cohorts suggest that immune checkpoint inhibitors can achieve clinically meaningful responses in a subset of patients with LMM (59). Both anti-PD-1 monotherapy and combination ipilimumab plus nivolumab have demonstrated activity, although response rates are lower than those observed in parenchymal brain metastases (60).

Combination immunotherapy appears to offer higher response rates than monotherapy, at the cost of increased toxicity. In a nationwide Danish study, patients with leptomeningeal disease had a median overall survival of 8.4 months, with systemic therapy associated with superior outcomes, though no survival benefit was seen with immune checkpoint inhibitors compared to BRAF/MEK inhibitors (61).

When interpreting these immunotherapy outcomes, it is important to recognize that intracranial radiological response and clinical benefit are frequently discordant in leptomeningeal disease. Response to immune checkpoint inhibitors should therefore be judged using the composite leptomeningeal criteria discussed above, which combine neurological status, CSF cytology, and imaging, rather than imaging alone. Corticosteroid dependence remains a major limiting factor, as steroid use is consistently associated with inferior outcomes (62, 63).

### Intrathecal immunotherapy

Intrathecal administration of immunotherapeutic agents represents an innovative strategy to overcome the limited penetration of systemic drugs into the CSF (63). Early-phase clinical trials and case reports have explored intrathecal interleukin-2 (64) and, more recently, intrathecal nivolumab alone (63, 65) or in combination with ipilimumab (66).

*A* phase I study of concurrent intrathecal and intravenous nivolumab demonstrated acceptable safety and signs of clinical and radiological activity in heavily pretreated patients with leptomeningeal metastases, including melanoma (63). With a median follow-up of 4.6 months, median overall survival was 7.0 months, with overall survival rates of 66.6% at 3 months, 53.1% at 6 months, and 34.8% at 12 months. The recommended intrathecal dose of nivolumab is 50 mg (with intravenous nivolumab 240 mg) every 2 weeks, and this approach has been incorporated into NCCN guidelines as a category 2B recommendation for melanoma patients patients with leptomeningeal involvement on stable or decreasing doses of dexamethasone (≤4 mg) (63).

The rationale for selecting a 50 mg dose, as well as the necessity of maintaining concomitant intravenous infusion, remains poorly defined. As with other monoclonal antibodies, nivolumab demonstrates rapid clearance from the cerebrospinal fluid (CSF) into the systemic circulation (65). Maintaining stable CSF concentrations can be achieved using implantable continuous infusion pumps (65). However, the relevance of maintaining stable nivolumab concentrations in the CSF is not known as it remains unclear whether a dose–response relationship exists for intrathecal nivolumab therapy.

The high clearance rate from the CSF to the blood also raises questions about the need for concomitant intravenous administration in patients receiving intrathecal nivolumab.

Finally, the optimal route of administration, lumbar catheter versus intraventricular catheter, has not been established. In the setting of leptomeningeal disease, impaired CSF circulation may theoretically favor intraventricular delivery to improve drug distribution within the intracranial subarachnoid space. Nevertheless, the risks associated with ventricular catheter placement must be carefully weighed, particularly in patients with lesions adjacent to the ventricular system, where the risk of hemorrhage may be increased. In published series of intrathecal therapy for leptomeningeal disease (LMD), many patients experience rapid clinical deterioration shortly after treatment initiation. It remains unclear whether starting intrathecal therapy earlier in the course of LMD could improve clinical outcomes.

However, these approaches remain investigational and should be pursued within clinical trials whenever possible.

### Targeted therapy in leptomeningeal disease

For patients with BRAF-mutant melanoma, BRAF and MEK inhibitors may induce rapid clinical improvement in LMM, particularly in symptomatic patients. Case series have reported transient responses and symptom relief, often facilitating corticosteroid tapering (67).

However, responses are typically short-lived, reflecting the emergence of resistance and limited durability of targeted therapy in the leptomeningeal compartment. Targeted therapy may therefore serve as a bridging strategy rather than a definitive treatment.

The agents used in this setting are the same BRAF/MEK inhibitor doublets approved for extracranial disease, namely dabrafenib plus trametinib, encorafenib plus binimetinib, and vemurafenib plus cobimetinib. Their activity in leptomeningeal disease is constrained by limited and variable penetration across the blood–CSF barrier and by active drug efflux, which may yield subtherapeutic CSF concentrations. For the small subset of patients whose tumours harbour actionable alterations beyond BRAF V600, such as NTRK fusions or KIT mutations, CNS-active agents (for example larotrectinib or entrectinib for NTRK fusions) may be considered, although evidence in leptomeningeal melanoma is anecdotal (68). Given the predictable development of resistance, BRAF/MEK inhibition in leptomeningeal disease is best positioned as a means of achieving rapid symptom control and corticosteroid sparing while a more durable immunotherapy-based strategy is established.

### Radiotherapy in leptomeningeal melanoma

For leptomeningeal metastases, craniospinal irradiation is rarely feasible due to toxicity and limited benefit. Focal radiotherapy may be used for symptom control in cases of bulky nodular leptomeningeal disease causing focal neurological deficits. In patients with very focal leptomeningeal disease, focal SRS can be discussed. It remains unclear whether prior irradiation can enhance the effectiveness of intrathecal (IT) immunotherapies. Whole-brain radiotherapy or craniospinal irradiation is generally avoided due to toxicity and lack of demonstrated survival benefit (69).

Palliative radiotherapy may also be used to alleviate symptoms and improve quality of life, particularly in patients with refractory pain or neurological deficits.

### Surgery

Surgical intervention has a very limited role in leptomeningeal melanoma metastases due to the diffuse nature of the disease. Surgery may be considered in exceptional cases for focal complications, such as hydrocephalus requiring shunting (communicating hydrocephalus) or ventriculocisternostomy (non-communicating hydrocephalus), or biopsy in cases of diagnostic uncertainty (70).

### Supportive care and symptom management

Optimal management of LMM requires meticulous supportive care. Control of intracranial pressure, seizure management, pain control, and psychological support are essential components of care. Early involvement of palliative care teams is strongly recommended, given the high symptom burden and poor prognosis (18). The corresponding management algorithm for leptomeningeal melanoma metastases is summarized in **Figure 2**.

**Figure 2:**
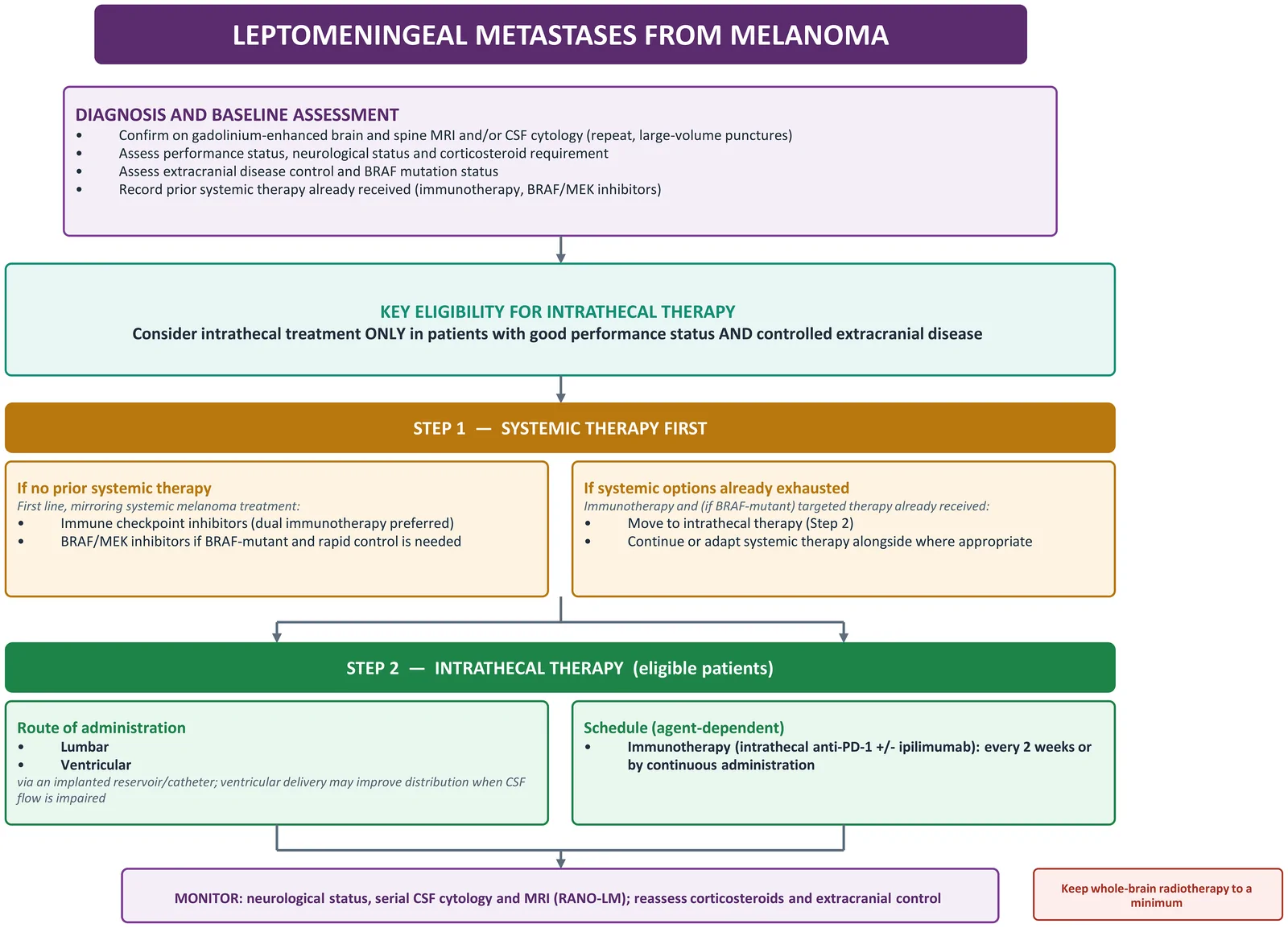
Management algorithm for leptomeningeal melanoma metastases (LMD), stratified by symptom status and BRAF mutation status. BRAF, v-Raf murine sarcoma viral oncogene homolog B; CSF, cerebrospinal fluid; ICI, immune checkpoint inhibitor; LMD, leptomeningeal disease; NGS, next-generation sequencing; WBRT, whole-brain radiotherapy.

## Emerging therapies and ongoing clinical trials

### Next-generation immune checkpoint inhibitors

Despite the success of current immune checkpoint inhibitors, primary and acquired resistance remains common, particularly in patients with CNS and leptomeningeal disease. Novel immune checkpoints targeting LAG-3, TIGIT, and TIM-3 are under active investigation and may enhance antitumor immunity within the CNS.

Relatlimab, an anti-LAG-3 antibody combined with nivolumab, has demonstrated efficacy in advanced melanoma, although specific data in patients with brain or leptomeningeal metastases remain limited. Ongoing trials are increasingly incorporating patients with stable or untreated brain metastases, reflecting a shift toward more inclusive trial designs (71).

### Cellular therapies and adoptive T-cell approaches

Adoptive cell therapies, including tumor-infiltrating lymphocytes (TILs) and genetically engineered T-cell therapies, represent a promising frontier for melanoma treatment. Early reports suggest that TIL therapy can induce responses in patients with brain metastases, although logistical complexity and toxicity remain significant challenges (72).

The applicability of cellular therapies to leptomeningeal disease is largely unexplored but conceptually attractive, given their ability to traffic to the CNS. Dedicated trials evaluating CNS-specific efficacy are needed.

### Targeting the tumor microenvironment and the blood–brain barrier

Novel strategies aimed at modulating the tumor microenvironment and improving drug delivery across the blood–brain and blood–CSF barriers are under development. These include small molecules with enhanced CNS penetration, antibody–drug conjugates, and nanoparticle-based delivery systems (73).

In parallel, anti-angiogenic agents and therapies targeting astrocyte–tumor interactions are being explored to disrupt the supportive niche that facilitates melanoma growth within the CNS (74).

### Biomarkers and liquid biopsies

The identification of predictive and prognostic biomarkers for CNS melanoma remains an unmet need. In melanoma with brain metastases, plasma ctDNA is often undetectable or poorly correlated with intracranial disease activity, which limits its utility for detecting or monitoring CNS progression, although undetectable ctDNA still carries favorable prognostic value for overall survival. By contrast, tumor DNA in cerebrospinal fluid can reliably reflect the molecular profile and dynamics of brain or leptomeningeal metastases in melanoma, suggesting CSF-derived cfDNA as a more informative compartment for CNS-focused biomarker work (75, 76).CSF-based molecular profiling may also provide insights into resistance mechanisms and guide personalized therapeutic strategies. Prospective validation of these biomarkers is ongoing.

### Practical clinical challenges and unmet needs

Several challenges recur in day-to-day management. First, patients with symptomatic disease, corticosteroid dependence, or leptomeningeal involvement are systematically underrepresented in pivotal trials, so much practice relies on extrapolation and retrospective data. Second, corticosteroids, often unavoidable for mass effect or radiation necrosis, blunt immunotherapy efficacy, creating tension between symptom control and optimal systemic treatment, and argue for early local therapy and rapid steroid tapering. Third, response interpretation is difficult: pseudoprogression, radiation necrosis, and discordant intracranial versus extracranial responses can each be mistaken for treatment failure, and standardized response criteria for leptomeningeal disease are insufficiently used. Fourth, the hemorrhagic propensity of melanoma metastases complicates decisions about anticoagulation, antiplatelet therapy, and the timing of radiosurgery. Finally, intrathecal therapy, continuous-infusion devices, and high-quality neuro-imaging are not uniformly available, and effective care depends on close coordination within a neuro-oncology multidisciplinary team. Addressing these gaps will require trial designs that deliberately include CNS-affected patients, CNS-specific endpoints, and validated liquid-biopsy biomarkers.

## Conclusion

The treatment of melanoma with cerebral and leptomeningeal involvement has significantly evolved in the last ten years. Previously uniformly lethal with few treatment alternatives, melanoma brain metastases are now possibly manageable in a significant number of patients, especially those with asymptomatic disease receiving combination immunotherapy and targeted radiation (50).

Prognosis of patients with symptomatic brain metastases remains, however, markedly limited. Similarly leptomeningeal disease remains an unmet medical need. The biological uniqueness of leptomeningeal disease marked by widespread CSF distribution, immune evasion, and restricted medication penetration, certainly accounts for the poor effectiveness of existing therapies (77–79).

Numerous significant issues arise from the current literature. Patients exhibiting symptomatic CNS disease, corticosteroid dependence, or leptomeningeal involvement are inadequately represented in clinical trials, hence constraining the generalizability of the findings. Secondly, the appropriate sequencing and mix of local and systemic therapy continue to be unknown, especially in individuals with BRAF-mutant melanoma (80).

Notwithstanding these hurdles, significant indicators of advancement are surfacing. Systemic immune checkpoint drugs can elicit sustained responses in a subgroup of individuals with leptomeningeal melanoma, challenging the historical pessimism associated with this illness. Intrathecal immunotherapy, while still experimental, presents a logical and novel strategy for surmounting pharmacological obstacles within the cerebrospinal fluid compartment (64). Advances in molecular profiling and liquid biopsy methodologies provide novel prospects for early diagnosis, treatment assessment, and individualized therapy.

From a therapeutic standpoint, the treatment of CNS melanoma necessitates a multidisciplinary and tailored strategy (81). Treatment decisions must incorporate neurological status, disease burden, molecular attributes, and patient preferences, necessitating strong collaboration among dermatologists, medical oncologists, neuro-oncologists, radiologists, radiation oncologists, and neurosurgeons. Such an approach is only feasible with regular radiological surveillance and systematic review of brain MRI by experienced neuroradiologists in close collaboration with neurosurgeons. Prompt referral to specialized centers and participation in clinical trials should be vigorously advocated, especially for individuals with leptomeningeal disease.

Given the heterogeneity of CNS melanoma, personalized multimodal strategies are increasingly advocated. Treatment decisions should be guided by neurological symptoms, disease burden, molecular profile, performance status, and patient preferences, ideally within a multidisciplinary tumor board setting (81).

Future research priorities encompass the advancement of CNS-penetrant treatments, the strategic formulation of combination regimens, and the integration of CNS-specific endpoints in clinical trials. Enhancing outcomes for individuals with leptomeningeal melanoma metastases necessitates both biological understanding and novel clinical trial designs that transcend traditional exclusion criteria.

In summary, although the outlook for melanoma with central nervous system involvement has significantly improved, especially concerning brain metastases, leptomeningeal disease continues to represent a significant unmet clinical need. Ongoing translational research, therapeutic innovation, and interdisciplinary collaboration will be crucial to enhance care.
